# Microenvironmental Analysis and Control for Local Cells under Confluent Conditions via a Capillary-Based Microfluidic Device

**DOI:** 10.1021/acs.analchem.2c02815

**Published:** 2022-11-16

**Authors:** Nobutoshi Ota, Nobuyuki Tanaka, Asako Sato, Yigang Shen, Yaxiaer Yalikun, Yo Tanaka

**Affiliations:** † Center for Biosystems Dynamics Research (BDR), RIKEN, 1-3 Yamadaoka, Suita, Osaka565-0871, Japan; ‡ Graduate School of Science and Technology, Nara Institute of Science and Technology, 8916-5 Takayama-cho, Ikoma, Nara630-0192, Japan

## Abstract

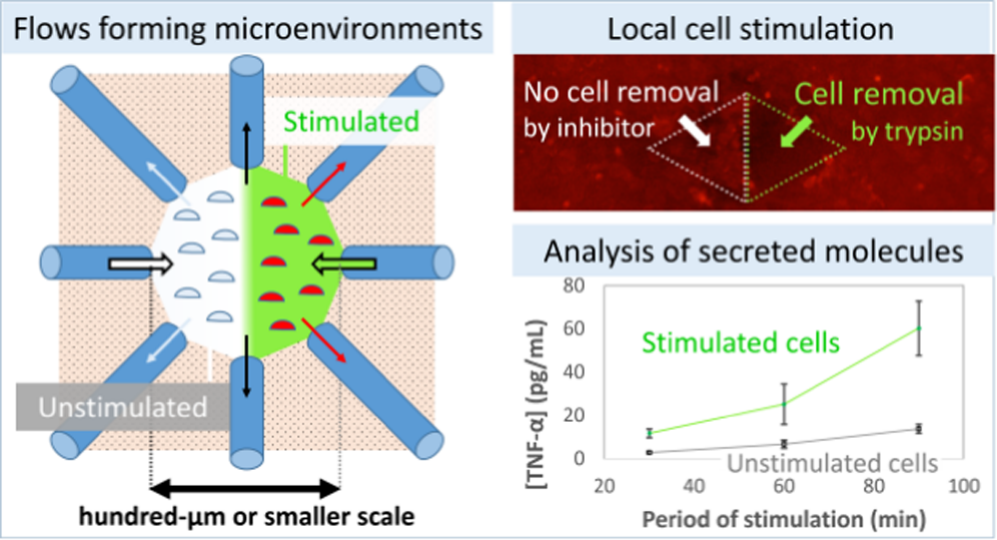

Sophisticated functions of biological tissues are supported by small biological units of cells that are localized within a region of 100 μm scale. The cells in these units secrete molecules to form their microenvironment to play a vital role in biological functions. Various microfluidic devices have been developed to analyze the microenvironment but were not designed for cells in a culture dish in a confluent condition, a typical setup for cell and tissue cultivation. This study presents a novel glass capillary-based microfluidic device for studying confluent cells in a culture dish. The multiple capillaries allow the device to confine the local flow in 100 μm or smaller scale to form two adjacent regions with different chemical properties; it can simultaneously perform local cell stimulation and collect secreted molecules from the stimulated cells. Cell removal was achieved upon trypsin stimulation from a limited area (3.8 × 10^–3^ ± 1.0 × 10^–3^ mm^2^), which corresponded to 7.6 ± 2.0 cells, using the mouse skeletal myoblast cell line (C2C12 cells) in a confluent condition. Microenvironmental analysis was demonstrated by measuring the secreted tumor necrosis factor alpha (TNF-α) collected from the microenvironment of the stimulated and unstimulated mouse leukemic monocyte cell line (RAW264 cells) to track temporal changes in the TNF-α production. The TNF-α secreted from stimulated cells was approximately four-fold higher than that from unstimulated cells in 90 min. This device enables local cell stimulation and the collection of secreted molecules for cells under confluent conditions, which contributes to the analysis of the cellular microenvironment.

Sophisticated functions of biological tissues and organs are supported by biological units of cells that are often localized in a small region of 100 μm scale, in which several tens to hundreds of cells reside on a plane.^1,2^ These biological units form their microenvironment through the molecules secreted from the constituent cells. Microenvironments enable communication with neighboring functional units and support the functions of a higher structure. For example, the subunits of the suprachiasmatic nucleus release peptide signals to their microenvironment to maintain a circadian rhythm.^3^ Another example is pathological tissues such as early-stage tumors localized on a 100 μm scale that also use their microenvironment to communicate with their neighbors to avoid attack from the immune system and convert healthy cells into pathological ones.^4^ A microenvironment is also a crucial analytical target to study developmental biology using tissue models such as organoids.^5^ Therefore, developing techniques for analyzing and controlling microenvironments is highly desired to understand and regulate the mechanisms used for tissue formation, organ function, control of pathological states, and the development of organisms.

Diverse methods have been reported to analyze cells and secreted molecules directly related to the microenvironment. The cellular analysis is well established and frequently employed to investigate the origins of microenvironments based on cellular molecules such as metabolites, cytoskeletal structures, and cell membranes. These molecules can be detected from homogenized or fixed cells^6−8^ and from the cell content that is suctioned out by a tiny needle^9,10^ for transcriptomic, proteomic, and metabolic analyses. However, these sampling methods are invasive and challenging to apply for the temporal analysis of the microenvironment. Labeling molecules of interest is another popular approach to visualize molecules, such as transporting vesicles^11^ and cellular receptors^12^ related to the microenvironment, although labeling is limited to a few known molecules. Hence, the diverse types of molecules in the microenvironment are challenging to track in biological samples. In contrast, the direct analysis of secreted molecules in the microenvironment can achieve temporal analysis of the cellular environment molecules ranging from small molecules such as nitrogen oxide^13^ and reactive oxygen species^14^ to large molecules such as cytokines.^15^ Direct analysis is also applicable for analyzing secreted molecules from a cultured functional unit, such as insulin from the pancreatic islets.^16^

Various methods have been developed to collect secreted molecules in the microenvironment. In addition to conventional microscale techniques, such as pipetting the liquid of the microenvironment directly,^17^ microfluidic devices have been widely used for collecting secreted molecules in the microenvironment through a microchannel for a medium supply of cells and tissues.^16,18^ In addition, these devices can provide complex conditions for culturing cells, for example, by applying two streams of distinct molecules in a microchannel to create a gradient in culturing conditions.^19,20^

However, the microfluid devices in previous reports were not designed for cells in a conventional culture dish. Hence, cells are cultured in a microchannel with an environment different from that of a culture dish. It has been reported that the mechanical properties of containers influence cell behaviors, cell morphology, cell differentiation, and gene expression.^21−23^ Unlike in a culture dish, a large portion of cells in a microfluidic device is located close to the microchannel walls and cells are influenced by the nearby walls. In addition, microchannels typically provide a small, isolated environment for culturing cells, making it challenging for large tissues to fit into these small spaces and there is an insufficient or slow supply of gas and nutrients. However, it is challenging to analyze the microenvironment of cells in a culture dish because it is difficult to separate the local liquids, such as those used for cell stimulation and the other liquids containing secreted cellular molecules, from the rest of the liquid in a dish. Although several devices have been reported to control local liquids and manipulate cells in conditions such as those present in a culture dish, these devices are not applied for analysis of microenvironmental molecules secreted from cells.^24−28^ Modifying the area of the local flow owing to the fixed position of the microfluidic entrance is also a complex process. Furthermore, these devices form spaces with closed tops and bottoms when the device approaches the bottom of a dish, thereby interfering with the supply of gas and nutrients for cells. Therefore, a fluidic device that is compatible with a conventional culture dish and is open for gas and nutrient supply is desirable for analyzing and controlling the microenvironment of cells in confluent conditions.

This study reports a capillary-based microfluidic device that is applied to cells under confluent conditions. This device can adopt varied positions, numbers, and sizes of the glass capillaries to provide a local flow matching the area of the target cells in a culture dish. In addition, the local flow creates stimulated and unstimulated cells in adjacent regions on a 100 μm scale. Therefore, the device used in this study is suitable for local cell stimulation and the analysis of the microenvironment of confluent cells in a culture dish.

## 2 Experimental Section

### 2.1 Chemicals and Materials

#### 2.1.1 Chemicals

Deionized water was generated using a TW-300RU system (Nomura Micro Science, Kanagawa, Japan). Antibiotics (penicillin–streptomycin, 100 folds, 168-23191) solution, trypsin ethylenediaminetetraacetic acid (trypsin–EDTA, 10 folds, 208-17251) solution without phenol red, nonessential amino acids (NEAAs, 100 folds, 139-15651), fluorescein (FL, 065-00252), lipopolysaccharide (LPS, 125-05201), and Dulbecco’s phosphate-buffered saline (D-PBS) (10 folds, 048-29805) were purchased from Fujifilm Wako Chemicals (Osaka, Japan). Fetal bovine serum (FBS) (FB-1290/500) was purchased from Biosera (Nuaille, France). The CellTracker Orange from Thermo Fisher (Waltham, MA), Dulbecco’s modified Eagle’s medium (DMEM) with phenol red (D6046), DMEM without phenol red (D4947), and DMEM-high glucose (D6429) were purchased from Merck (Kenilworth, NJ).

#### 2.1.2 Materials

A mouse skeletal myoblast (C2C12) cell line (RCB0987) and mouse leukemic monocyte (RAW264) cell line (RCB0535) were purchased from the RIKEN BRC Cell Bank (Ibaraki, Japan). The plastic dishes (Falcon 353002) were purchased from Corning (Corning, NY). The 96-well plates (92096) were purchased from TRP (Trasadingen, Switzerland). The glass capillaries with a polyimide coating (inner diameter (id) of 40, 180, or 250 μm, outer diameter (od) of 100 or 360 μm) were purchased from Molex (Lisle, IL).

#### 2.1.3 Fabrication of Capillary Devices

The capillary device consisted of 3 parts: glass capillaries, capillary holder, and xyz stage (TSD-605CL, Sigma Koki, Saitama, Japan). The capillary holders were fabricated using a three-dimensional (3D) printer using clear resin (Mr. Glad Factory, Osaka, Japan). The capillary holder had 15 mm diameter and held capillaries at 45° angle from a dish surface. This design was aimed to apply for culture dishes with 35 mm or larger diameters although this holder can be applied for a well with smaller diameter if its design is modified (Table S1). The polyimide coating on the capillary edge was removed to avoid any fluorescence induced by an excitation wavelength of 480 nm. Pipette tips stabilized the positions of the glass capillaries set on the capillary holder, and PDMS caps were placed on the holder.

#### 2.1.4 Simulation of Fluidic Control

Single-liquid-phase fluidic simulation with fluid–solid interaction was performed using the volume of fluid (VOF) laminar flow model to analyze the flow speed of the microflow near capillaries and cells. A flow model including the inlet and outlet capillaries was created using Rhino software (Robert McNeel & Associates, Seattle, WA). Simulations with the finite volume method were implemented using computational fluid dynamics software (SimFlow, Simflow Technologies, Warsaw, Poland). For the convenience of calculations, we used simple simulation conditions in which the surfaces of the dish and capillaries were fixed layers while other surfaces were set as open boundaries. The inlets and outlets were set as the mass flow inlets and outlets. The inlet and outlet flow conditions were 3.0 and 20.2 μL/min per capillary, respectively. The flow speed profiles were obtained using ParaView open software (Kitware Inc.).

The flow rate was confirmed by observing the flow of fluorescent beads (Fluoresbrite YG Microspheres, Polysciences, Warrington, PA) in PBS containing 10% FBS. Glass capillaries (id/od = 180/360 μm) provided a flow of 1 μm beads from the right inlet and 2 μm beads from the left inlet. Movies of the flowing beads were recorded using a charged-coupled device (CCD) camera. The movies were converted to images (15 frames/s) by the Python program to analyze the flow speed using JPIV open software (Github) for particle image velocimetry.

#### 2.1.5 Cell Cultivation

C2C12 cells were cultured in a plastic dish containing D6429 supplemented with 10% FBS and the antibiotics. The RAW264 cells were cultured in a plastic dish containing D6046, 10% FBS, and 0.1% NEAA. Each cell type was cultured at 37 °C and 5% CO_2_. For the RAW264 cultivation, FBS was heat inactivated at 56 °C for 30 min before transferring to the culture medium. For the local cell removal experiments, the C2C12 cells were cultured under confluent conditions. For the local cell stimulation experiments, RAW264 cells were cultured until the cell density reached nearly confluent conditions.

#### 2.1.6 Local Removal of C2C12 Cells

For the local cell treatment, the capillary device was placed above the cultured cells to provide microflow that was controlled using a syringe pump (Fusion-400, Chemyx, Stafford, TX) for the inlet capillaries and hydrodynamic pressure for the outlet capillaries. For the cell analysis, the cultured cells were observed under a microscope (IX71, Olympus, Tokyo, Japan) equipped with excitation/emission wavelengths of 480/520 nm for fluorescence observation or transmitted light for phase-contrast observation. The cells were maintained at 37 °C by placing them on a temperature-controlled plate (MATS-55R30, Tokai Hit, Shizuoka, Japan) on the microscope stage. The images were obtained using a CCD camera (DP-82, Olympus) connected to a computer for analysis using the software (cellSens, Olympus).

C2C12 cells were used in a culture dish to remove local cells under fully confluent conditions. Before the cell removal, the cells were fluorescently labeled with CellTracker at 37 °C after two rinses with PBS. After 30 min of incubation, the labeling solution was discarded. The labeled cells were rinsed twice with PBS which was then replaced with 6 mL of PBS with or without 10% FBS. The cells were treated with two opposing microflows of inlet capillaries to investigate the various conditions involved in local cell removal. One inlet capillary contained trypsin and 20 μM FL, and the other inlet was the negative control that contained no trypsin (with or without FBS). The introduced solutions were collected using the outlet capillaries. The images of green fluorescence provided by FL were used to visualize the trypsin distribution based on the microflow. The images of the red fluorescence using CellTracker and images of the transmitted light were used to analyze the cell removal. Cell viability was checked by labeling C2C12 cells with calcein-AM (for live cells) and propidium iodide (for dead cells), instead of labeling by CellTracker, via a labeling kit (CS01, Dojindo, Kumamoto, Japan) by following manufacture’s instruction. At excitation light of 460–495 nm and emission light of >510 nm, calcein-AM in live cells strongly fluoresced green and propidium iodide weakly fluoresced red. Therefore, live cells (green) and dead cells (red) were observed simultaneously. Positions of dead cells were checked by excitation light of 530–550 nm and emission light of >570 nm to fluoresce only propidium iodide.

#### 2.1.7 Local Stimulation of RAW264 Cells and Analysis of Secreted Tumor Necrosis Factor (TNF-α)

For the local stimulation of the RAW264 cells, the same instrumental setup described in the previous section was employed. Before cell stimulation, the cells were rinsed twice by D4947 with 10% FBS, 0.1% NEAA, 100-fold diluted antibiotics stock solution, and no phenol red (hereafter referred to as transparent medium), followed by replacing with 6 mL of the fresh transparent medium. Local cell stimulation was performed by opposing the microflow from the two inlet capillaries (id/od = 180/360 μm). One flow contained 100 ng/mL LPS and 20 μM FL in the transparent medium. The other flow, which contained no LPS or FL in the transparent medium, served as a negative control. The introduced solutions and secreted cellular TNF-α were collected via the outlet capillaries in 0.2 mL plastic tubes. The images of green fluorescence provided by the FL were used to analyze the LPS distribution based on the microflow. The images of the transmitted light were used to count the number of stimulated cells. TNF-α and FL were quantified using an enzyme-linked immunosorbent assay (ELISA) kit for mouse TNF-α (RSD-MTA00B-1, R&D Systems, Minneapolis, MN) according to the manufacturer’s instructions using a spectrophotometer (FLAME-S-UV–VIS–ES, Ocean Insight, Orlando, FL).

To check the influence of LPS and antibiotics on TNF-α secretion of RAW264 cells, confluent RAW264 cells in a 96-well plate were used. Influence of antibiotics was investigated by comparing TNF-α secretion of RAW264 cells incubated in 100 μL of culture medium (D4947 with 10% FBS and 0.1% NEAA) with or without antibiotics for 0–3 h at 37 °C and 5% CO_2_ to collect supernatant. The medium with antibiotics was the same as the transparent medium. Influence of LPS in various concentrations was investigated by comparing TNF-α secretion of RAW264 cells incubated in 100 μL of the transparent medium with 0, 0.01, 0.05, 0.1, 0.5, 1, or 10 μg/mL of LPS for 0.5 h at 37 °C and 5% CO_2_ to collect supernatant. After incubation, 80 μL of supernatant was collected from each well to quantify the concentration of secreted TNF-α by the ELISA kit and the spectrophotometer.

## 3 Results and Discussion

### 3.1 Characterization of the Capillary Device

**Figure 1:**
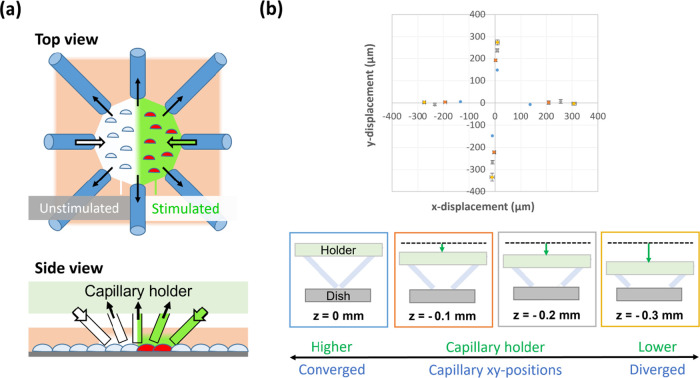
Experimental setup of device and control of capillary positions. (a) Illustrations of device use. Microflow controlled by glass capillaries forms adjacent areas of stimulated and unstimulated cells and collects liquid of the cellular microenvironment. (b) Capillary positions controlled by the relative height (*z*) of the capillary holder from the surface of the dish. (Top) Positions of four capillaries with standard deviation (*n* = 3). Blue: *z* = 0 mm, red: *z* = −0.1 mm, gray: *z* = −0.2 mm, and yellow: *z* = −0.3 mm. (Bottom) Illustrations for positioning capillaries by adjusting the distance between dish surface and capillary holder of device. When four capillaries converged at the center, *z* was determined as 0. By lowering the capillary holder, the capillaries were farther apart.

The capillary device used in this study was designed to control and collect liquid from the cellular microenvironment (Figure 1a); the actual device is illustrated in Figure S1. As illustrated in Figure 1b, the surface of the dish set the *z* positions of the capillary tips, and the *xy* positions of the capillaries were controlled by the *z* position of the capillary holder. The *z* position of the capillary holder from the dish was determined as a relative height (*z*) of 0 mm when the capillary tips touched each other at the center (that is, when the distance between the capillaries was minimized to converge all capillaries). The capillaries settled in the dispersed positions as the capillary holder was lowered to decrease the relative height. This correlation may be described by eq 1
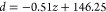
1where, *d* (μm) is the distance of the capillary position from the center and *z* (μm) is the relative height. Hence, the capillary distance from the center was determined linearly by the relative height.

**Figure 2:**
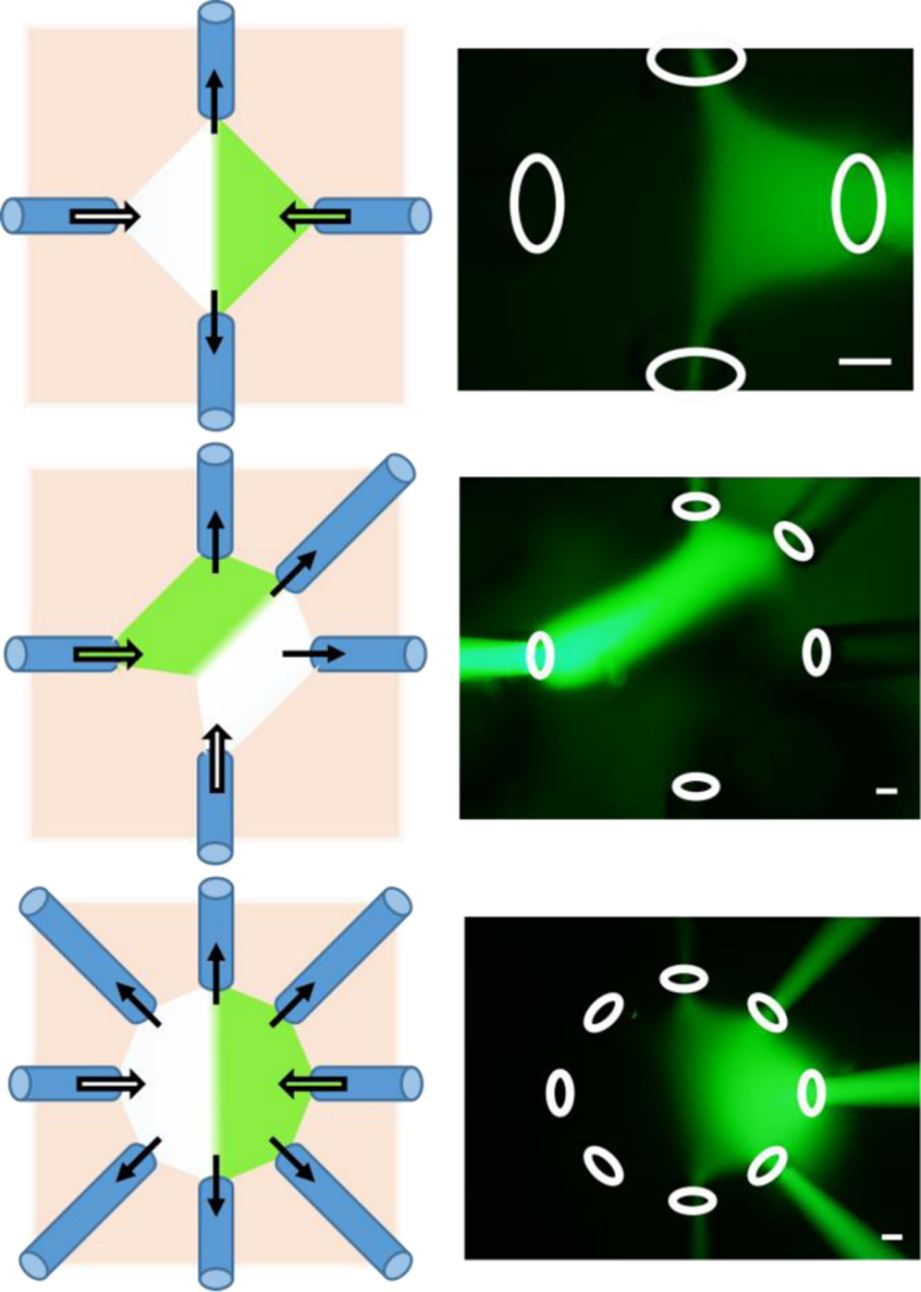
Flow pattern, stability, and simulation of capillary-based flow. Flow patterns formed by the present device. Illustrations in the left column show capillary positions and two regions formed by two types of introduced liquid. Arrows indicate flow directions. Pictures in the right column show the actual liquid flow. White circles indicate open ends of glass capillaries. Scale bar: 100 μm.

The device can create multiple patterns and areas of flow by positioning the capillaries at the desired positions. Figure 2 illustrates three flow patterns. Among these patterns, the stability of the flow was investigated for the setup of four and eight capillaries. For the setup of four capillaries, opposing capillaries were set 600 μm apart. Each inlet capillary was connected to a syringe, and a syringe pump controlled the flow rate at 3.0 μL/min. The right inlet provided the flow visualized by FL, and the left inlet provided the flow with no visible color. Each outlet capillary was placed in a container and collected liquid at a flow rate of 20.2 μL/min. The total flow rates of all inlets and all outlets were 6.0 and 40.4 μL/min, respectively, and the ratio of outlet/inlet flow rates was 6.7. A stable flow was observed for 10 min in this condition (Figure S2a). The same capillary and flow conditions were used for further experiments with the four-capillary setup unless otherwise noted. For the setup of eight capillaries (two inlets and six outlets), opposing capillaries were also set 600 μm apart. Minimum inlet and outlet flow rates per capillary were 1.5 and 2.2 μL/min, respectively, to observe stable flow (Supporting Video 1). The total flow rates of all inlets and all outlets were 2.0 and 10.4 μL/min, respectively, and the ratio of outlet/inlet flow rates was 4.4. A stable flow was observed for 2 h in this condition.

The minimum ratio of outlet/inlet flow rates is 1 in theory to drain all stimulating chemicals introduced from the inlets. In contrast, the ratios in the setups above were found to be more than 1 to compensate diffusion of the introduced chemicals and offset the sudden increase of introduced solutions caused by pulsating motion of a syringe pump. Meanwhile, an optimized ratio of outlet/inlet flow rates varies depending on number and dimensions of equipped capillaries as well as distance between the capillaries. The above setups showed a stable FL region with no apparent overflow for 10 min or longer by having more than 4 for the ratio of outlet/inlet flow rates. In addition, it was observed that the right and left inlet flows formed the middle border between the fluorescent and nonfluorescent regions. Therefore, the flow controlled by the capillaries could effectively maintain the stimulated and unstimulated regions.

A simulation was performed for the flow controlled by the four capillaries (Figure S2b). In this simulation, the opposing capillaries were placed 500 μm apart, and all of the capillaries were positioned 200 μm above the bottom of the dish. The flow velocities were 1.0 mm/s for each inlet and 6.9 mm/s for each outlet, corresponding to flow rates of 3.0 and 20.2 μL/min, respectively. The simulation results showed that the opposing flows from the right and left inlets met to form a border along the line connecting the two outlets. At the height of the capillaries, the minimum flow velocity was approximately 0.5 mm/s at the middle of the two inlets where the opposing flows met, although the flow velocities were mainly found to be 1.0 mm/s or more for other regions. In contrast, the flow velocities were found at 1.0 mm/s or less at the height of the dish. This simulation result was verified by an experiment in which fluorescent beads showed a similar tendency of the flow velocities (Figure S2b).

### 3.2 Chemical Conditions Influencing Cell Removal

**Figure 3:**
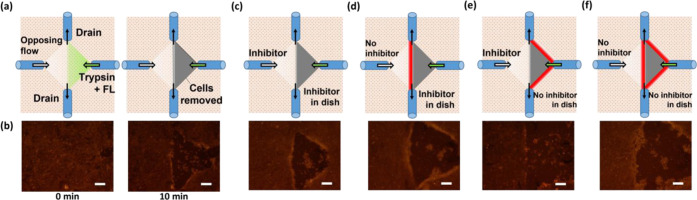
Local removal of C2C12 cells in the culture dish with confluent conditions controlled by trypsin and trypsin inhibitor. (a) Illustrations of applied flow pattern and resulting cell-removed area. (b) Pictures of stained C2C12 cells. (Left) Beginning of cell removal (0 min) and (right) cell removal at 10 min. (c–f) Cell removal by trypsin flow from right with different conditions of opposing flow and bulk liquid. Red areas are susceptible to active trypsin diffusion because trypsin flow met regions containing no trypsin inhibitor. (c) Trypsin inhibitor contained in opposing flow and bulk liquid in the dish. (d) PBS flow and trypsin inhibitor contained only in bulk liquid in the dish. (e) Trypsin inhibitor in opposing flow and no trypsin inhibitor in bulk liquid. (f) No trypsin inhibitor in opposing flow and bulk liquid in the dish. Scale bar: 100 μm.

Cell removal was performed on a myoblast cell line (C2C12 cells) using a four-capillary experimental setup to investigate local cell control by the microenvironments formed via capillary flow (Figures 3a,b and S3). Inlet and outlet flow rates were 3.0 and 20.2 μL/min per capillary, respectively. Outlet flow rate was set larger than inlet flow rate to compensate diffusion of the chemicals introduced from the inlets. The right inlet introduced a microflow of trypsin (0.5 w/v%) in PBS, which was visualized by FL. This trypsin solution had a trypsin concentration of a stock solution that was 10-fold higher than that typically employed (0.05 w/v%) for removal of C2C12 cells in the cell passage. The viability of collected cells was 95.4 ± 2.0% (*n* = 6) after treatment of 0.5% trypsin and passing through capillaries of the device. Figure S4 shows typical results of the stained C2C12 cells. This concentration was selected to determine whether trypsin diffusion occurred by applying a trypsin microflow. The left inlet introduced a microflow of PBS with (Figure 3c,e) or without trypsin inhibitor (Figure 3d,f) contained in FBS. The middle border was formed by the opposing flows from the right and left inlets. The middle border appeared distinct because the trypsin inhibitor interfered with the diffused trypsin when the trypsin inhibitor was introduced from the left inlet (Figure 3c). In addition, the trypsin digestion was limited to regions near the trypsin flow (Figure S3) because the outlet capillaries drained the inlet flow and bulk liquid from the surrounding area.

Consequently, this limitation prevented the diffusion of trypsin toward the outside of the targeted region for cell removal. When PBS excluding the trypsin inhibitor was introduced from the left inlet, the middle border of cell removal occurred slightly more toward the PBS side than in the middle between the trypsin and PBS flows, owing to competition for trypsin diffusion and the opposing PBS flow (Figure 3d). Trypsin digestion did not occur in most cells under PBS flow, even though no trypsin inhibitor was included in the PBS flow.

Similarly, the bulk solution in a cell culture dish was investigated to determine its effect on cell removal. When PBS containing the trypsin inhibitor was added to a dish, cell removal occurred with indistinct borders between the trypsin flow and the bulk environment, although a distinct border of cell removal was found in the middle of the trypsin flow and opposing flow (Figure 3e). The borders of cell removal appeared indistinct when no trypsin inhibitor was contained in the opposing flow and bulk PBS. This occurrence was observed although cell removal was limited to a finite area due to competition between trypsin diffusion and the slow flow toward the outlets (Figure 3f). Overall, the combination of trypsin and trypsin inhibitors enabled the removal of local cells. These results indicate that the manipulation of local cells could be achieved by selecting an appropriate combination of chemical conditions for stimulation and competition.

### 3.3 Physical Conditions Influencing Cell Removal

**Figure 4:**
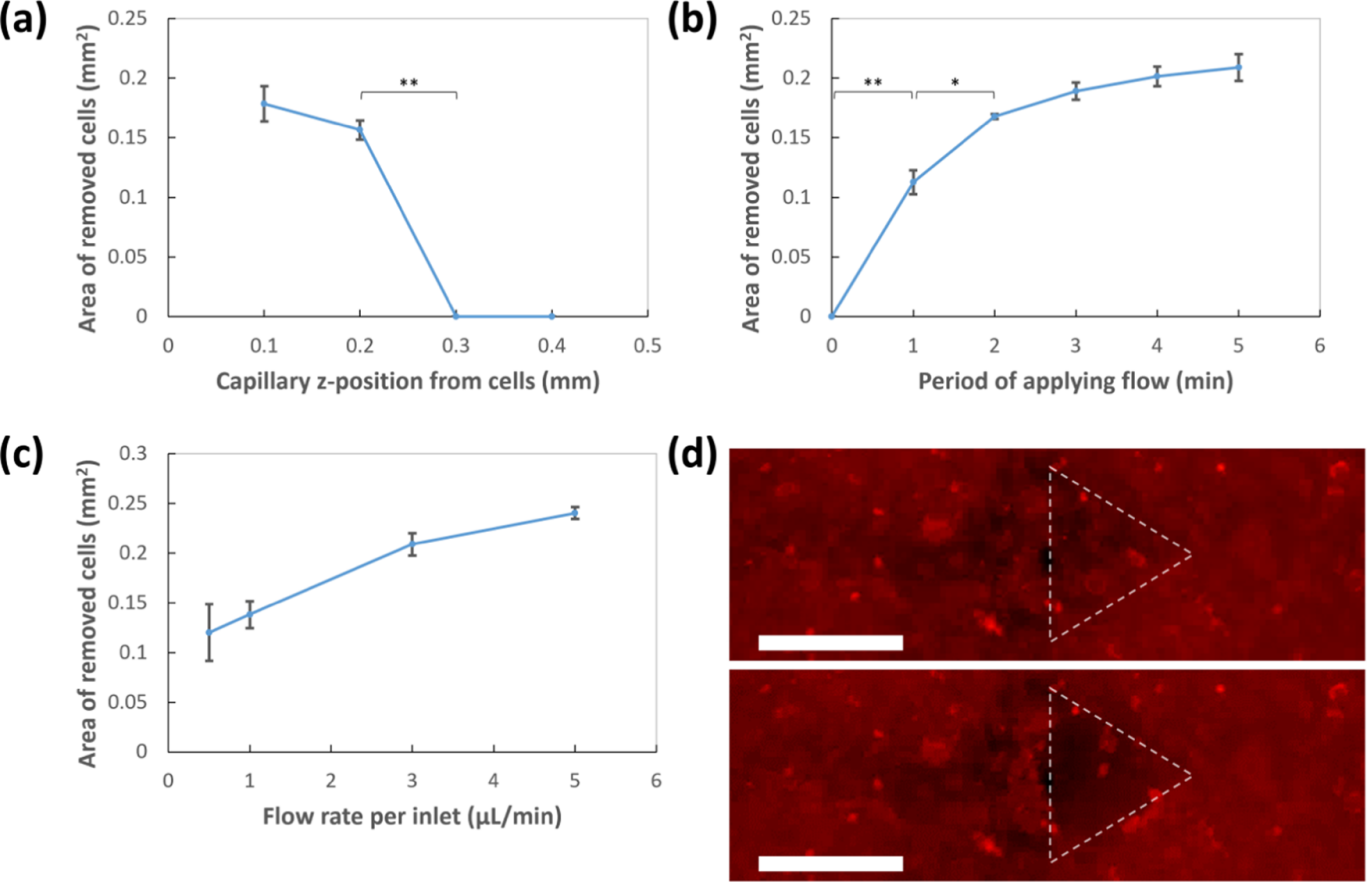
Removal of C2C12 cells influenced by physical factors. Area of cell removal influenced by (a) inlet and outlet ends of flow, (b) period of applying flow, and (c) flow rate. *: *p* < 0.05; **: *p* < 0.01. (d) Removal of small number of cells in a confluent condition using capillaries with an id and od of 40 and 100 μm. (Top) Before and (bottom) 2 min after applying trypsin flow. Enclosed area is where cells were removed. Brightness and contrast of images were modified for cell visibility. Scale bar: 100 μm.

Besides the influence of chemical conditions, cell removal was also influenced by the microflow conditions, namely, the *z* position of capillaries controlling the inlet and outlet ends of the microflow, period of applied flow, and flow rate (Figure 4). C2C12 cells were treated with trypsin flow and opposing flow containing trypsin inhibitor to investigate these conditions. Four capillaries were used to control the cellular microenvironment. PBS containing trypsin was introduced from the right inlet for cell removal. The PBS containing the trypsin inhibitor was introduced from the left inlet to prevent trypsin digestion of the cells as a negative control. The introduced solutions were drained from the two outlets. The flow rate was 3.0 μL/min for each inlet unless otherwise noted. The outlet flow rate was fixed at 20.2 μL/min for each outlet.

First, the cell removal was investigated based on the *z* positions of the glass capillaries controlled by the xyz stage of the device. The *z* positions were determined from the bottom edge of the capillaries by setting the height of the C2C12 cells in a culture dish to a relative height of 0 mm. Local cell removal occurred when the bottom of the capillaries was within 200 μm from the cells at the bottom of the culture dish (Figure 4a). When the capillaries were set to 300 μm or above from the cells, the cells remained in the region of the right inlet flow, indicating that trypsin could not reach the cells. The capillary *z* positions were set at 150 μm from the cells for further investigation.

Thereafter, the effect of the period of applying trypsin flow on the cell removal was investigated at an inlet flow rate and outlet flow rate of 3.0 and 20.2 μL/min, respectively. As shown in Figure 4b, the area of cell removal increased rapidly in the first 2 min. Subsequently, the change in the area became much smaller after 3–5 min of applying trypsin flow. Based on these results, a duration of 5 min was selected for further investigation.

The influence of the trypsin flow rate on cell removal was investigated by introducing trypsin solution at flow rates of 0.5, 1.0, 3.0, and 5.0 μL/min on the right inlet. The same flow rate of the trypsin inhibitor was applied from the left inlet while the flow rate was fixed at 20.2 μL/min for each outlet. A faster inlet microflow rate increased the area of cell removal because the trypsin flow, as visualized by FL, could spread over a larger area (Figure 4c). An inlet flow rate faster than 5 μL/min was challenging to stabilize and resulted in trypsin digestion of the cells in the undesired cell regions at a fixed outlet flow rate. Overall, the area of cell removal was controlled by these physical parameters through the device and glass capillaries.

In addition to microflow conditions, the capillary size is also an essential factor in controlling the area of cell removal because the positions of the capillaries are influenced by their size. Using four glass capillaries with small diameters (id/od = 40/100 μm) set to the same capillary holder, opposing capillaries were placed 100 μm apart. A low flow rate was applied with precise control using hydrodynamic pressure. The right and left inlets provided trypsin and trypsin inhibitor, respectively. Each inlet applied the solution to C2C12 cells at a flow rate of 10 nL/min from 100 μm above the cells. The introduced solution was drained from the outlet capillaries at a rate of 20 nL/min for each outlet. These capillary and flow conditions were selected to perform small-area cell removal in a short period. To illustrate a representative result of the cell removal for this experimental setup, Figure 4d shows pictures emphasizing areas of before and after trypsin treatment (Figure S5 shows the whole pictures of those in Figure 4d). These pictures were modified in contrast (+65%) and brightness (+30%) to distinguish the difference of cell-present and cell-removed areas easier. The cell removal occurred in an average area of 3.8 × 10^–3^ ± 1.0 × 10^–3^ mm^2^ (*n* = 5), although the borders of cell removal needed more careful investigation, comparing with those in Figure 3, due to a lower signal-to-noise ratio in observation at high magnification. The estimated number of removed cells was 7.6 ± 2.0 cells based on the average number of cells (approximately 2000 cells/mm^2^) at this confluent condition. Although staining cellular nuclei was desired to count removed cells with high accuracy, resin used in the present device was not compatible to work with a fluorescent dye excited by UV wavelengths. Hence, the present device can be improved by exploring alternative materials for the device component to allow a wider range of excitation wavelengths. Overall, stable cell removal was achieved by controlling the chemical and physical conditions.

### 3.4 Measurements of TNF-α Secreted from Locally Stimulated Cells

The studied device simultaneously stimulated the cells of interest and collected chemicals secreted from the stimulated cells using the appropriate chemical and physical parameters. This function was useful for creating and monitoring a locally unique group of cells. In addition, the number of capillaries employed could be easily changed by this device owing to its simple structure.

**Figure 5:**
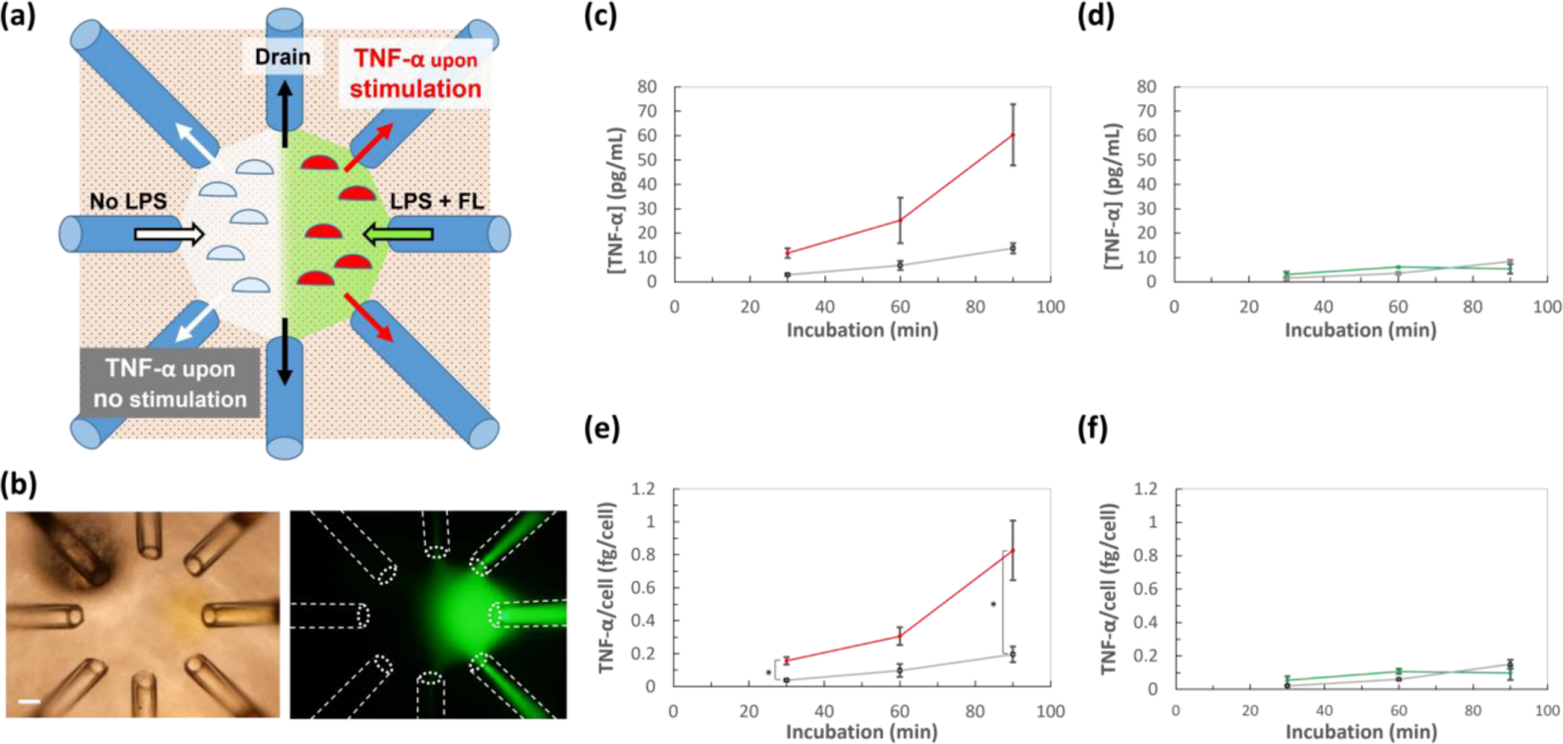
Secreted TNF-α obtained from the microenvironment of stimulated and unstimulated cells. (a) Illustration of simultaneous operation of stimulating local cells and collecting secreted molecules in the microenvironment. (b) Images of simultaneous operation. (Left) Capillary positions. Right inlet was for LPS introduction and left inlet was for culture medium (no LPS) introduction. Upper and lower right outlets collected liquid from the right inlet which passed above LPS-stimulated cells. Upper and lower left outlets collected liquid from the left inlet which passed above unstimulated cells. Center outlets drained liquid from both right and left inlets. Scale bar: 100 μm. (Right) LPS flow visualized by FL. Dotted lines indicate capillary positions. (c, d) Calculated TNF-α concentrations for cells in right and left regions. (c) TNF-α concentrations for LPS-stimulated cells in the right region (red) and unstimulated cells in the left region (gray). (d) TNF-α concentrations for unstimulated cells in right (green) and left (gray) regions. Panel d is a negative control of panel c. (e, f) Calculated TNF-α production per cell for cells in right and left regions. *: *p* < 0.05. (e) TNF-α/cell for LPS-stimulated cells (red) and unstimulated cells (gray). (f) TNF-α/cell for unstimulated cells in right (green) and left (gray) regions. Panel f is a negative control of panel e.

Mouse leukemic monocyte (RAW264) cells were used to investigate the capability of the device to stimulate local cells and collect chemicals secreted from locally stimulated cells (Figure 5). For this purpose, RAW264 cells were stimulated by LPS, a chemical that initiates the response of monocytes to bacterial cells, to induce the secretion of TNF-α (Figure 5a). The device was equipped with eight capillaries (two inlets and six outlets). Each opposing pair of capillaries was 600 μm apart. The right inlet introduced LPS solution to create a stimulated group of cells. The LPS solution contained FL to visualize the regions of the stimulated cells. The left inlet introduced the culture medium to create an unstimulated group of cells. Each inlet introduced a solution at 1.0 μL/min. Two outlets were placed approximately 200 μm away from the right inlet to collect TNF-α, which was mainly derived from the stimulated cells. Two outlets were placed 200 μm away from the left inlet to collect TNF-α, which was mainly derived from unstimulated cells. The other outlets were used to drain the liquid introduced from the two inlets. Each outlet drained solution at 2.6 μL/min. The ratio of outlet/inlet flow rates was 7.8, larger than the ratio in Supporting video 1, to minimize LPS diffusion so that LPS stimulation was limited to the cells only in a targeted area. If LPS diffusion occurred, undesired cells were stimulated to secrete TNF-α, which could result in an increased background signal of TNF-α.

The collected liquid was a mixture of liquid that passed above the target cells and from the area surrounding the target region because the total flow rate of the collected liquid (15.6 μL/min) was greater than the total flow rate of the introduced liquid (2.0 μL/min). As shown in Figure 5b, the LPS solution from the right inlet flowed above the stimulated cells to transfer TNF-α from the stimulated cells to the nearby outlets. Thus, the FL concentration in the collected sample was used to estimate the concentration of TNF-α derived from the stimulated cells before dilution by the liquid from the surrounding region, using eq 2
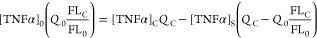
2where [TNF-α]_0_, [TNF-α]_C_, and [TNF-α]_S_ are the TNF-α concentrations (pg/mL) in the LPS-stimulated region before dilution, the collected sample, and the surrounding unstimulated region, respectively. *Q*_0_ and *Q*_C_ are the flow rates of the LPS solution (1 μL/min) and sample collection (5.2 μL/min), respectively. FL_0_ and FL_C_ are the FL quantities (pmol) in the introduced LPS solution and the collected sample, respectively.

TNF-α from the stimulated cells was collected at 30, 60, and 90 min of applying flow, followed by ELISA evaluation. Figure 5c,d illustrates the measured TNF-α concentrations in each region for the positive and negative controls, respectively. In addition, the number of cells in each region was found to be between 1500 and 1900 (Figure S6) to calculate TNF-α per cell (Figure 5e,f). The positive control had an LPS-stimulated region on the right and an unstimulated region on the left. The average TNF-α concentration with standard error for the 30, 60, and 90 min samples was calculated as 11.8 ± 2.0, 25.3 ± 9.4, and 60.3 ± 12.5 pg/mL for the LPS-stimulated region and 2.9 ± 0.2, 6.7 ± 1.8, and 13.8 ± 2.1 pg/mL for the unstimulated region. The corresponding TNF-α per cell was 0.16 ± 0.03, 0.31 ± 0.09, and 0.83 ± 0.32 fg/cell for the LPS-stimulated region and 0.04 ± 0.01, 0.10 ± 0.07, and 0.20 ± 0.09 fg/cell for the unstimulated region. The values of TNF-α per cell in the left and right regions were significantly different (*p* < 0.05) in 30 and 90 min samples (Figure 5e).

The negative control had no LPS stimulation in either the right or the left regions. In contrast, the TNF-α concentrations were similar in both regions. The calculated TNF-α concentrations for the 30, 60, and 90 min samples were 3.1 ± 1.1, 6.1 ± 0.1, and 5.4 ± 1.9 pg/mL for the right unstimulated region and 1.5 ± 0.7, 3.5 ± 0.5, and 8.3 ± 0.8 pg/mL for the left unstimulated region. The corresponding TNF-α per cell was 0.05 ± 0.03, 0.11 ± 0.02, and 0.10 ± 0.04 fg/cell for the right unstimulated region and 0.02 ± 0.01, 0.06 ± 0.01, and 0.15 ± 0.03 fg/cell for the left unstimulated region. The values of TNF-α per cell in the left and right regions were not significantly different in 30, 60, and 90 min samples (Figure 5f).

As the incubation period increased, the TNF-α production slowly increased in the under-flow and no-flow regions. The RAW264 cells cultured in the 96-well plates also exhibited a slow production of TNF-α with no LPS and a rapid increase in the TNF-α secretion upon exposure to LPS (Figure S7). Hence, the shear stress induced by the microflow was considered to have a negligible influence on the RAW264 cells in this study.

The TNF-α concentration and TNF-α/cell ratio of the LPS-stimulated cells were significantly higher than those of the unstimulated cells (Figure 5c,e). In addition, the TNF-α/cell values for the LPS-stimulated cells in this study were similar to those in a previous report.^29^ Overall, the studied device simultaneously prompted cell stimulation and collected the secreted chemicals to monitor temporal changes in the local cells of interest. This device is also applicable for analysis of other secreted molecules, and Table S2 summarizes measurable molecules that are secreted from RAW264 cells. As the present device with ELISA detection allowed to detect and quantify TNF-α of 1 pg/mL (60 pM) or larger scale, the molecules in Table S2 are also measurable in their reported concentrations in the 0.1–100 nM scale.^30−33^ Furthermore, this device produced adjacent stimulated and unstimulated cell groups, which simplified the comparison of the two groups by placing both groups in the same environment.

## 4 Conclusions

This study successfully demonstrated local cell stimulation and liquid collection from the cellular microenvironment using a capillary-based microfluidic device. The studied device could function in a culture dish with confluent cells and was able to selectively stimulate local cells among neighboring cells. The capillary setup was easily modified to stimulate cells in the range of approximately 10 to 2000 cells. In addition, unlike a closed microchannel, this device allowed the gas and nutrient supply to reach the cells easily because the capillaries kept the top side of the cultured cells open.

It has been challenging to form heterogeneous groups of cells in adjacent regions using previously reported methods in a conventional culture dish, and an unconventional platform, such as a microfluidic device, needed to be employed for this purpose. This situation has limited opportunities to analyze tissues and organs composed of heterogeneous functional units because artificially developed models can be made only on unconventional platforms. The studied device removed the current limitations of the microenvironmental analysis. Furthermore, the device was versatile and could be used to control the microenvironment of tissue and organ samples, form a heterogeneous group of cells from the same type of cells in a culture dish, and manipulate 3D-structured cellular spheroids and organoids through local stimulation. The device is also potentially applicable for molecular profiling, such as transcriptomics and metabolomics, for locally stimulated/unstimulated groups of cells. Glass capillaries have also been widely used for capillary electrophoresis and sample preconcentration. Thus, the present device can be directly applied to further chemical analysis, including analyzing the genetic materials of local cells removed from tissue by trypsin digestion. Therefore, the present device is applicable for a wide range of applications, which opens the possibility of microenvironmental analysis and engineering.

## Supporting Information Available

Experimental setup of device and control of capillary positions (Figure S1); comparison of simulation and experimental results of flow velocities (Figure S2); correspondence of trypsin flow and removal of C2C12 cells (Figure S3); viability of C2C12 cells after trypsin treatment and passing through capillaries (Figure S4); removal of small number of C2C12 cells (Figure S5); region of LPS-stimulated RAW264 cells (Figure S6); TNF-α released from RAW264 cells cultured in a 96-well plate (Figure S7); compatibility of the present device with various containers (Table S1); ELISA-detected molecules that are released from RAW264 cells upon stimulation by LPS or other materials (Table S2) (PDF)
Flow provided by 8 capillaries for 2 h (Video 1) (MP4)
