# Supplementary material for: Microenvironmental Analysis and Control for Local Cells under Confluent Conditions via a Capillary-Based Microfluidic Device
Source: Anal Chem. 2022 Nov 16;94(47):16299–307. doi: 10.1021/acs.analchem.2c02815 (PMC9716555; doi:10.1021/acs.analchem.2c02815)
Supplement: Supplementary file 1 — ac2c02815_si_001.pdf [file ac2c02815_si_001.pdf]

Supporting information

Microenvironmental analysis and control for local cells in a confluent condition via a capillary-based microfluidic device

Nobutoshi Ota,[1]* Nobuyuki Tanaka,[1] Asako Sato,[1] Yigang Shen,[1] Yaxiaer Yalikun,[1,2] and Yo Tanaka[1]*

[1]*Center for Biosystems Dynamics, RIKEN, Suita, Osaka 565-0874, Japan*

[2]*Graduate School of Science and Technology, Nara Institute of Science and Technology, Ikoma, Nara 630-0192, Japan*

*To whom correspondence should be addressed

Tel.: +81-6-6105-5132, FAX: +81-6-6105-5132

E-mail: nobutoshi.ota@riken.jp

Table of Contents:

- Supporting Figures, Tables and Legends (Figure S1-S7 and Table S1-S2)
- Supporting Movie Legends (Supporting Video 1)

This 12-page supporting information, including 7 additional figures, 2 tables and 1 video legend, intends to provide details of analytical conditions and experimental results more comprehensively.

Supporting figures

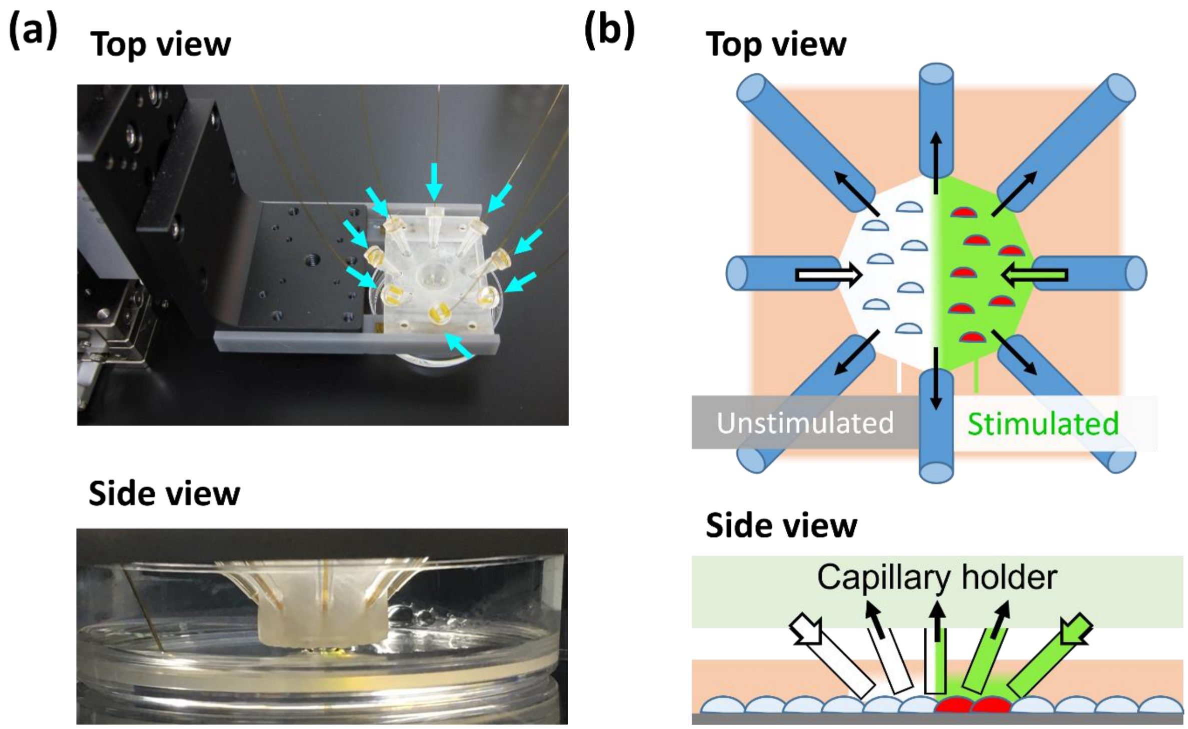

Figure S1. Experimental setup of device and control of capillary positions.

(a) Image of capillary device. (Top) Image of device consisting of glass capillaries, capillary holder, and xyz-stage. Arrows indicate where capillaries are attached to device. (Bottom) Picture of device set above cell culture. (b) Illustrations of device use. These illustrations are the same as Figure 1a.

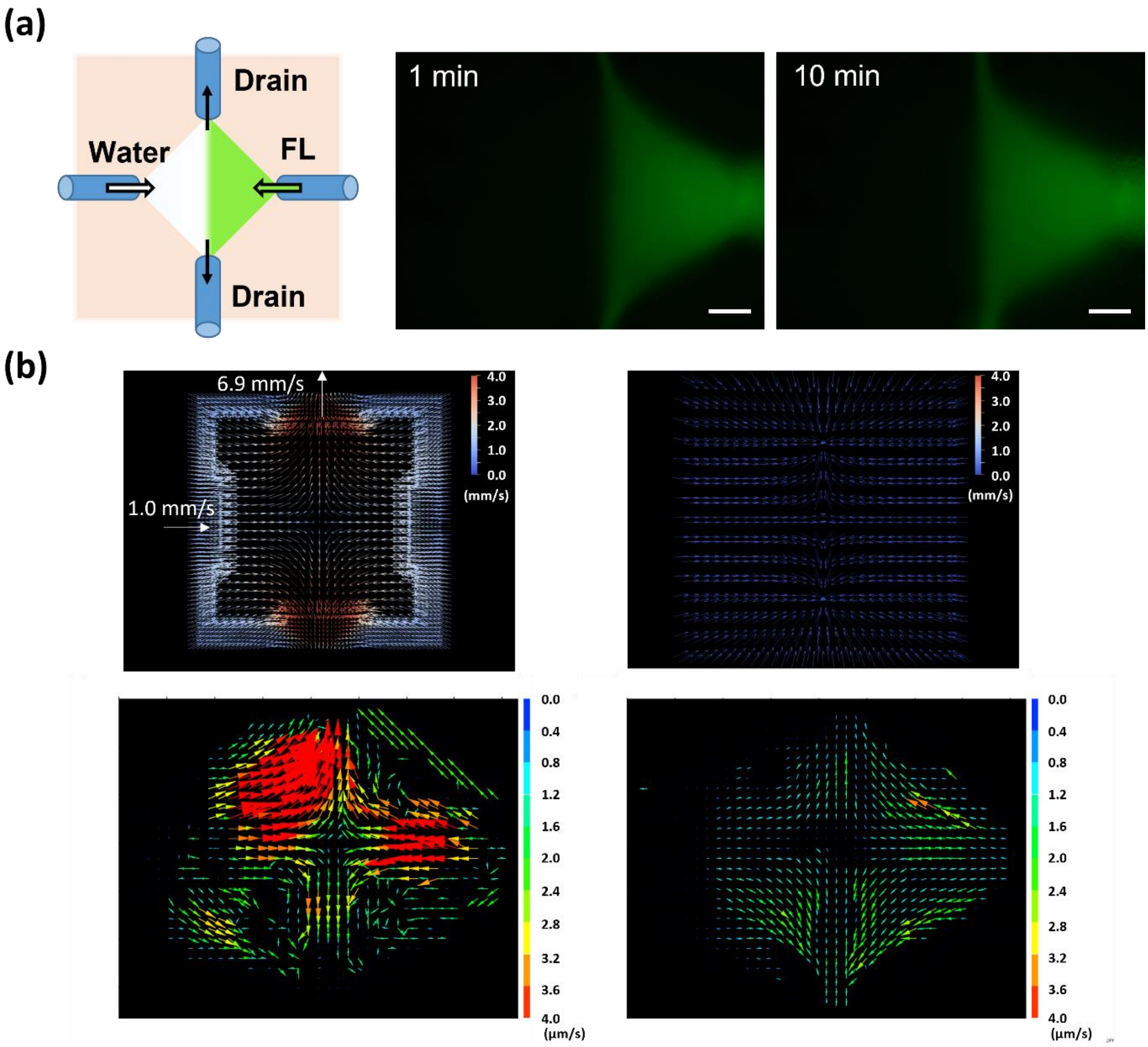

Figure S2. Comparison of simulation and experimental results of flow velocities.

(a) Illustration and pictures of trypsin flow visualized by 20 μM FL at 1 and 10 min of applying flow. Scale bar: 100 μm. (b) Flow velocities of 4 capillaries. (Top row) Calculated flow velocities by simulation at (left) 200 μm and (right) 40 μm above dish surface. Inlet and outlet flow velocities were set 1.0 and 6.9 mm/s, respectively, and corresponding flow rates were 3.0 and 20.2 μL/min, respectively. (Bottom row) Experimental flow velocities measured by flowing beads at the focal plane of (left) 200 μm and (right) 40 μm above dish surface.

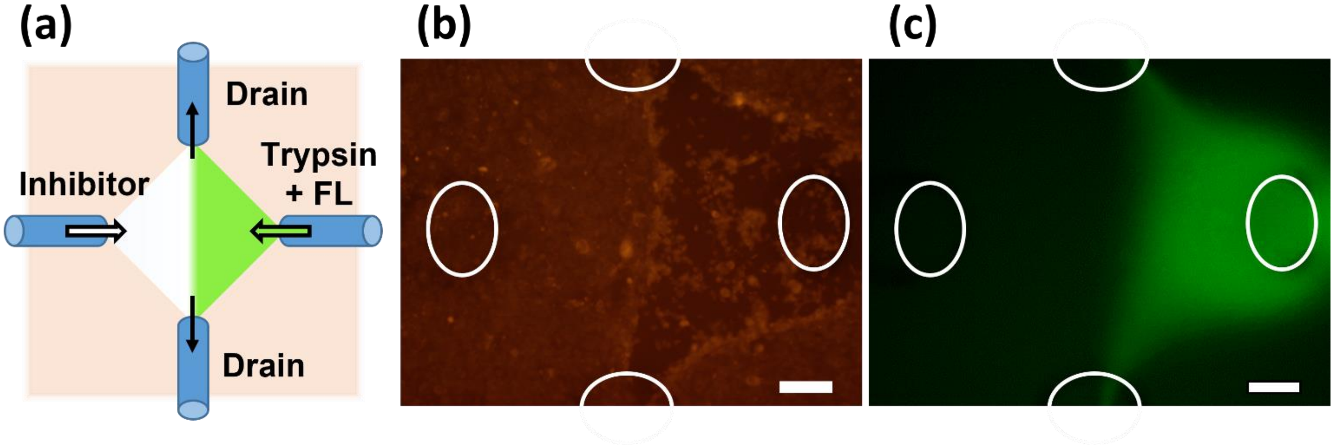

Figure S3. Correspondence of trypsin flow and removal of C2C12 cells.

(a) An illustration for the flow setup of trypsin, trypsin inhibitor, and fluorescein (FL) used to obtain images (b) and (c). (b) An image of removal of C2C12 cells stained by CellTracker Orange. (c) An image of trypsin flow visualized by 0.2 µM FL. This image was modified in brightness (+40%). (b) and (c) were obtained within 10 seconds after applying 2 min of trypsin flow from the right inlet. White circles indicate open ends of capillary inlets and outlets. Scale bar: 100 µm.

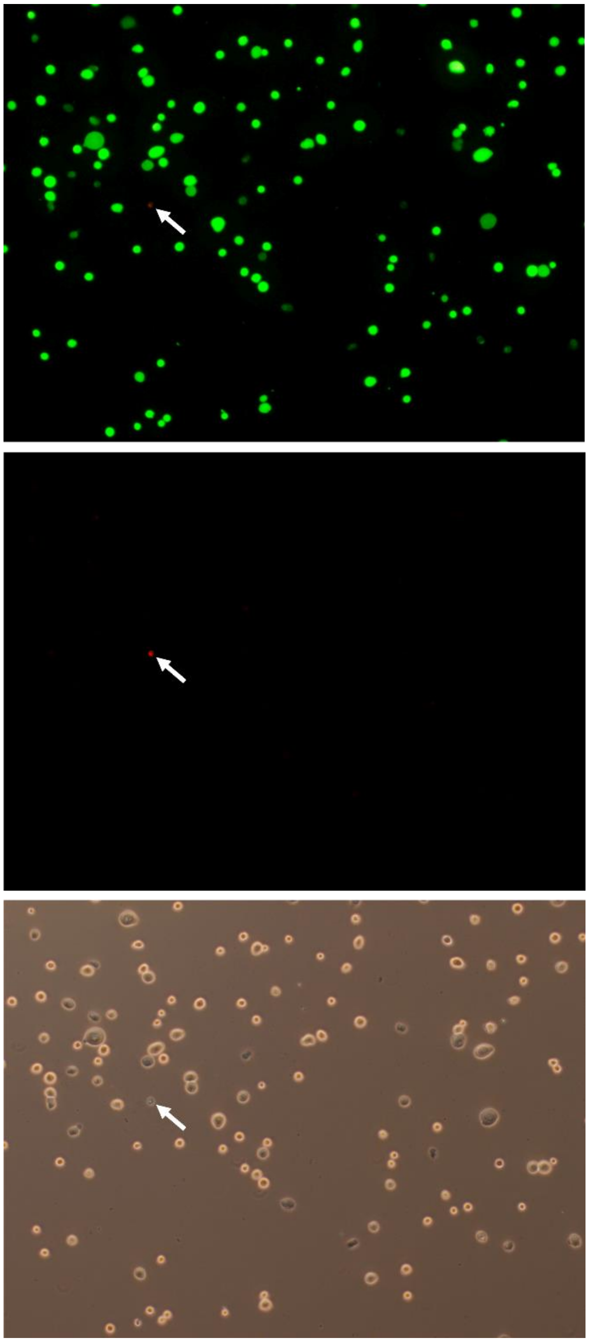

Figure S4. Viability of C2C12 cells after trypsin treatment and passing through capillaries.

(Top) Live C2C12 cells stained by calcein-AM (green) and dead C2C12 cells stained by propidium iodide (weak red) at excitation and emission wavelengths of 460-495 nm and >510 nm. (Middle) Dead C2C12 cell (red) at excitation and emission wavelengths of 530-550 nm and >570 nm. (Bottom) Bright field observation of the C2C12 cells. Arrows indicate the same dead cell stained only by propidium iodide that

fluoresced red by receiving excitation wavelengths of 460-495 nm or 530-550 nm.

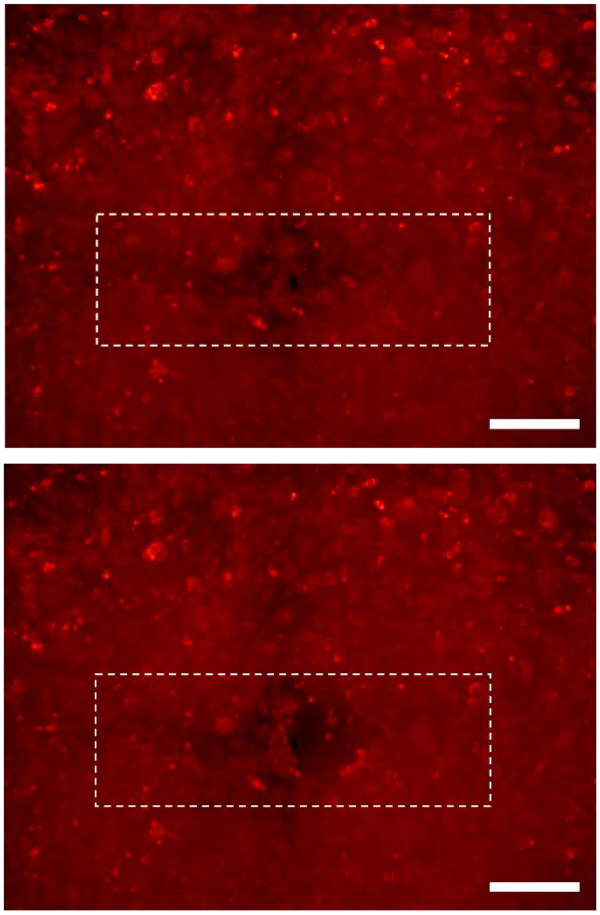

Figure S5. Removal of small number of C2C12 cells.

C2C12 cells (Top) before and (Bottom) 2 min after applying trypsin flow. Enclosed areas are shown in Figure 4. Scale bar: 100 μm.

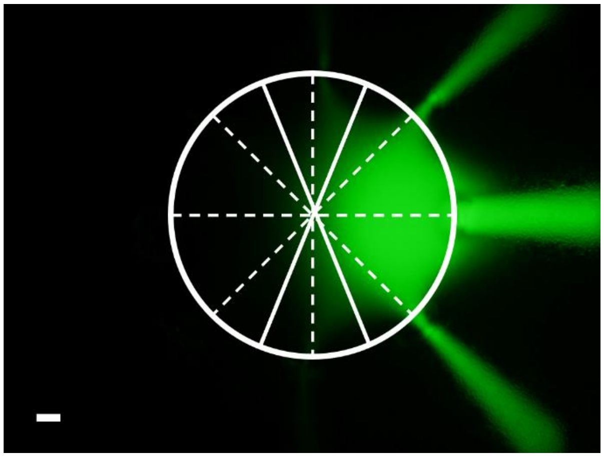

Figure S6. Region of LPS-stimulated RAW264 cells.

Region of LPS-stimulated cells was assigned from LPS flow visualized by fluorescein. LPS flow was introduced from right inlet. As shown in the picture above, majority of fluorescent liquid was drained by capillaries of upper and lower right and the rest of liquid was drained by center capillaries. Liquid collected by right capillaries was approximately obtained from three-eighth of the area enclosed by a white circle in the picture. Hence, number of the cells in this region was used to calculate TNFα per cell. Scale bar: 100 μm.

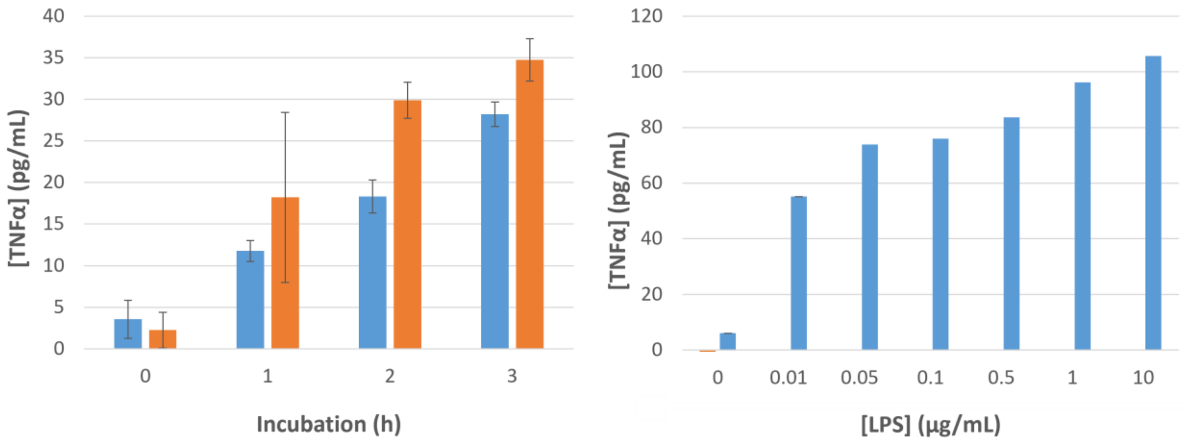

Figure S7. TNFα released from RAW264 cells cultured in 96-well plate.

(Left) TNFα release from RAW264 cells incubated in (blue) culture medium only and (red) culture medium with antibiotics for 0 to 3 hours. (Right) TNFα release from RAW264 cells incubated (blue) in culture medium containing various concentrations of LPS for 0.5 hours and (red) in culture medium with no LPS for 0 hour as a reference.

Container	Typical diameter of a dish/well (mm)	Typical depth of a dish/well (mm)	Compatibility with the present device
**35-mm dish**	**34.5-35**	**7-9-**	**Yes**
**6-well plate**	**34-35**	**17-18**	**Yes**
**12-well plate**	**22-23**	**17-18**	**Yes**
**24-well plate**	**12.5-14**	**12.5-16**	**No***
**96-well plate**	**5-7.5**	**10.5-11.5**	**No****

Table S1: Compatibility of the present device with various containers.

(* and **) The present device requires modification of the capillary holder to fit the device in a well. A modified holder needs to have its diameter less than the diameter of a target well. A modified holder also need to keep capillaries at an angle of ≧55 and ≧68 degree to fit a well of a 24- and a 96-well plate, respectively.

Secreted molecules detected by ELISA	Introduced materials other than LPS	References
IL-6 and IL-1β	Allomyrinasin	30
IL-1α and IL-6	Heat-treated *Lactobacillus crispatus* KT-11	31
IL-6, IL-12, and IL-10	*Cervus nippon var. mantchuricus* extract	32
IL-1β and PGE2	Marein	33

Table S2. ELISA-detected molecules that are released from RAW264 cells upon stimulation by LPS or other materials.

Abbreviations: interleukin (IL), prostaglandin E2 (PGE2).

Supporting video legends

Supporting video 1. Flow provided by 8 capillaries (2 inlets and 6 outlets) for two hours. Each inlet introduced solution at 1.5 μL/min and each outlet drained the solution at 2.2 μL/min. Right inlet introduced aqueous solution of 20 μM fluorescein. Left inlet introduced water. The ratio of outlet/inlet flow rates was 4.4 to stabilize flows.
